# Progress towards Operation of a Deuterium Cold Neutron Source at the NCNR

**DOI:** 10.1088/1757-899X/755/1/012025

**Published:** 2020-06

**Authors:** J Jurns, M Middleton, R Williams

**Affiliations:** 1 NIST Center for Neutron Research, Gaithersburg, MD 20899, USA

## Abstract

The NIST Center for Neutron Research (NCNR) operates a 20 MW research reactor that produces neutrons for a suite of 30 neutron scattering instruments. 70% of these instruments use cold neutrons (E<5 meV), which are moderated by two separate cold neutron sources. The cold moderator for both sources is liquid hydrogen (LH_2_), which is in turn cooled by a recently commissioned 7 kW, 14K helium refrigerator. NCNR plans to replace the larger cold source with a new one operating with liquid deuterium (LD_2_). This report focuses on progress towards the upgrade to liquid deuterium, and options to address the particular challenges of designing and operating a cooling system that simultaneously supports operation with both LH_2_ and LD_2_.

## 1. Introduction

The National Institute of Standards and Technology (NIST) Center for Neutron Research (NCNR) operates a 20 MW research reactor that produces neutrons for a suite of 30 neutron scattering instruments. 70 % of these instruments use cold neutrons (E < 5 meV), which are moderated by two separate cold neutron sources. The cold moderator for both sources is liquid hydrogen (LH_2_), which is in turn cooled by a recently commissioned 7 kW, 14K helium refrigerator. NCNR plans to replace the larger cold source with a new one operating with liquid deuterium (LD_2_). This report focuses on progress towards the upgrade to liquid deuterium, and options to address the particular challenges of designing and operating a cooling system that simultaneously supports operation with both LH_2_ and LD_2_

## 2. Background

The National Bureau of Standards Reactor (NBSR) is a 20 MW research reactor operated by the NCNR, primarily for neutron scattering instruments to study the properties of materials, but also for research in fundamental physics and nuclear chemistry. The first guide hall was completed in 1989 and the first liquid hydrogen (LH_2_) cold neutron source (CNS) was installed in 1995 [1]. In 2012, the NCNR completed a major expansion project, adding a new guide hall and 5 new guides. With the expansion of the facility, the cold neutron sources have been improved and expanded. The original LH_2_ source was replaced with the Advanced LH_2_ Cold Source (Unit 2) in 2002, doubling the flux of cold neutrons to all the instruments [2,3]. Additionally, a second LH_2_ source (“Peewee”) [4] was installed in thermal neutron beam port BT-9, solely for the Multi-Axis Crystal Spectrometer (MACS II) which was relocated to BT-9 to accommodate the 5 new guides. The only way to improve upon the optimized Unit 2 is to replace it with a large volume, liquid deuterium (LD_2_) source.

Future plans for the NBSR are to convert to low-enriched uranium (LEU) fuel [5]. The conversion to LEU will result in an anticipated loss of 10 % in neutron flux. The upgrade to LD_2_ is planned to mitigate this loss. Monte Carlo simulations show that a LD_2_ source provides an average gain in brightness of 1.5 between 4 Å and 9 Å with respect to the existing liquid hydrogen cold source, and a gain in brightness of 2 at the longest wavelengths. This conversion is being done with the support and funding from the National Nuclear Security Administration of the Department of Energy (DOE). Without this support from the DOE this project would not be possible.

## 3. Current configuration

The LH_2_ cold sources at NBSR are designed for simple, reliable operation. The overall design is very similar to the horizontal cold source in the High Flux Reactor at the Institut Laue-Langevin in Grenoble [6]. Hydrogen is liquefied in a condenser heat exchanger located approximately 2 m above the moderator. The LH_2_ is gravity fed to the moderator where neutrons are slowed as they pass through the LH_2_. Heat transferred to LH_2_ by the neutrons vaporizes a portion, which returns to the condenser to be re-liquefied. This results in a thermosiphon design that operates completely passively. The entire hydrogen system includes the moderator cryostat, condenser heat exchanger, interconnecting piping and a warm gas buffer tank. The system is hermetically sealed to insure its integrity, and there are no pressure relief devices of any kind. All components are surrounded by a blanket of helium to prevent the possibility of creating a mixture of hydrogen and air. Since the cold source is a passive design operator intervention is never required to maintain the safety of the system. Should cooling fail (refrigerator failure) the hydrogen simply boils off and is safely stored in the buffer tank. The reactor is shutdown to prevent over heating of the moderator vessels.

**Figure 1.**
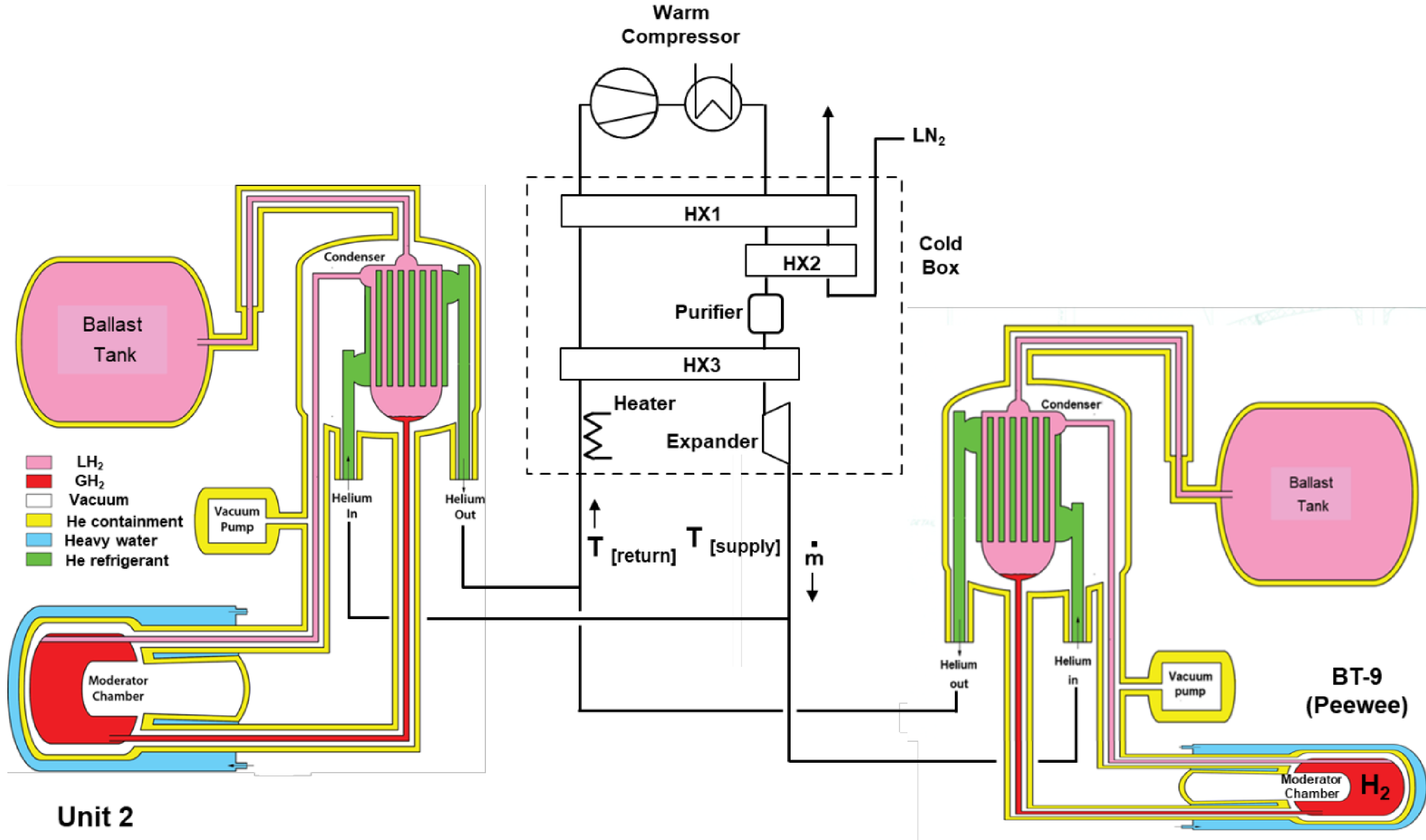
NBSR simplified cold source and helium refrigerator schematic diagram.

A simplified schematic of the two hydrogen cold neutron sources and helium refrigerator are shown in figure 1.

## 4. Planned changes

To achieve the gains anticipated by converting to LD_2_, a large volume (35 liters) of LD_2_ is required. The expected nuclear heat load in this moderator and vessel is 4 kW. To accommodate this increased heat load, a new, 7 kW helium refrigerator was installed and commissioned in 2018 to provide the necessary cooling capacity. The Unit 2 LH_2_ cold source will be removed, and the new LD_2_ cold source installed in its place. The moderator cryostat is designed to the same overall dimensions as Unit 2 and will fit in the existing reactor beam port. Table 1 shows the current and expected cold source heat loads.

**Figure 2.**
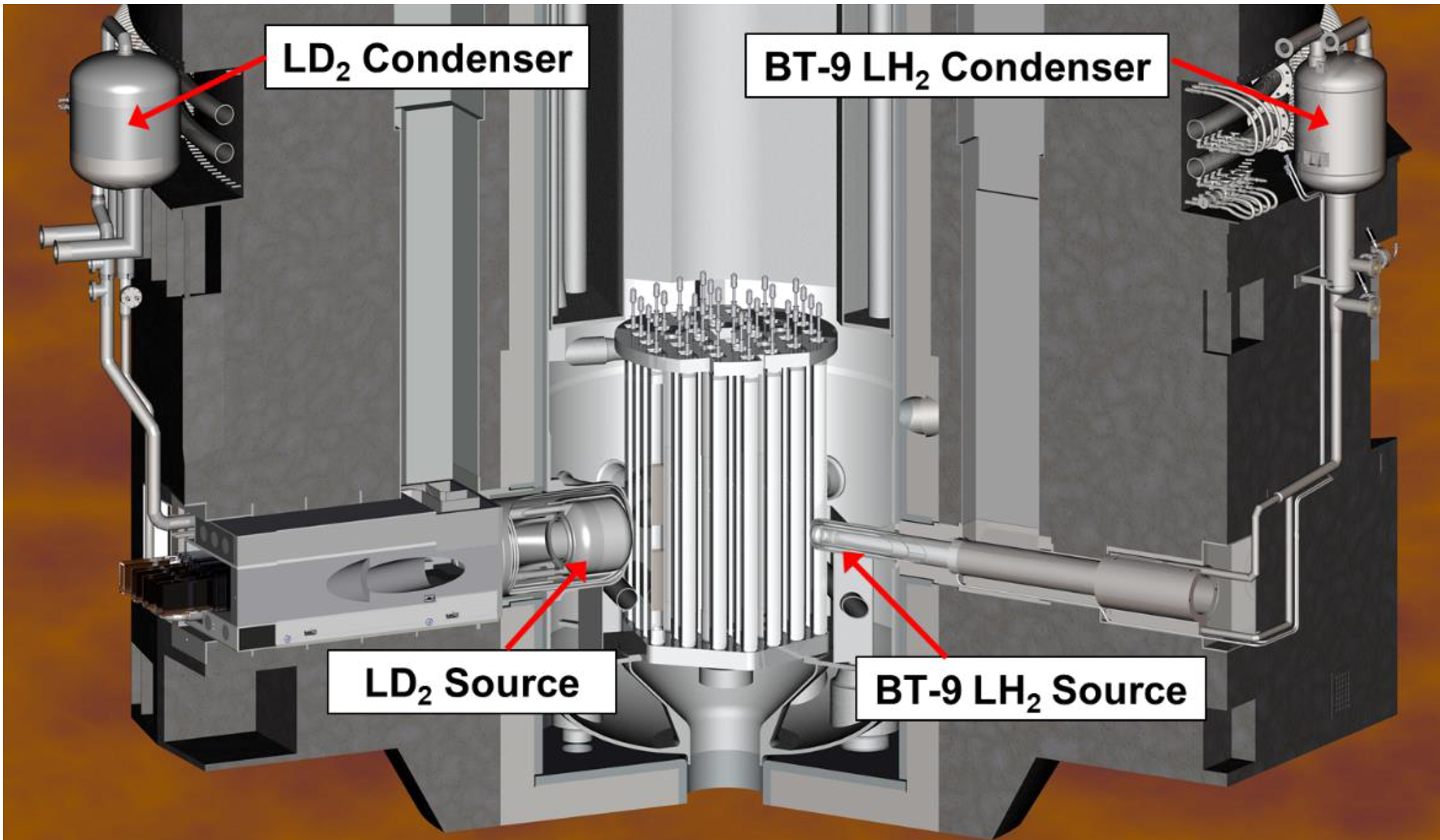
LD_2_ and Peewee LH_2_ cold sources in NBSR reactor

As with the existing LH_2_ cold sources, this source will operate as a naturally circulating thermosiphon. A condenser mounted on the reactor face approximately 2 m above the source provides the gravitational head to supply the source with LD_2_. The system will be open to a 16 m^3^ ballast tank to store the warm deuterium gas at 4–5 bar when the refrigerator is not operating, providing a passively safe response to a refrigerator trip. The source will operate at 23.3 K, the boiling point of LD_2_ at 100 kPa. All components will be surrounded by a helium blanket to prevent the possibility of creating an air/deuterium mixture. A cutaway model of the new LD_2_ cold source and existing Pewee cold source is shown in figure 2.

## 5. Design challenges – current operation with LH_2_

**Figure 3.**
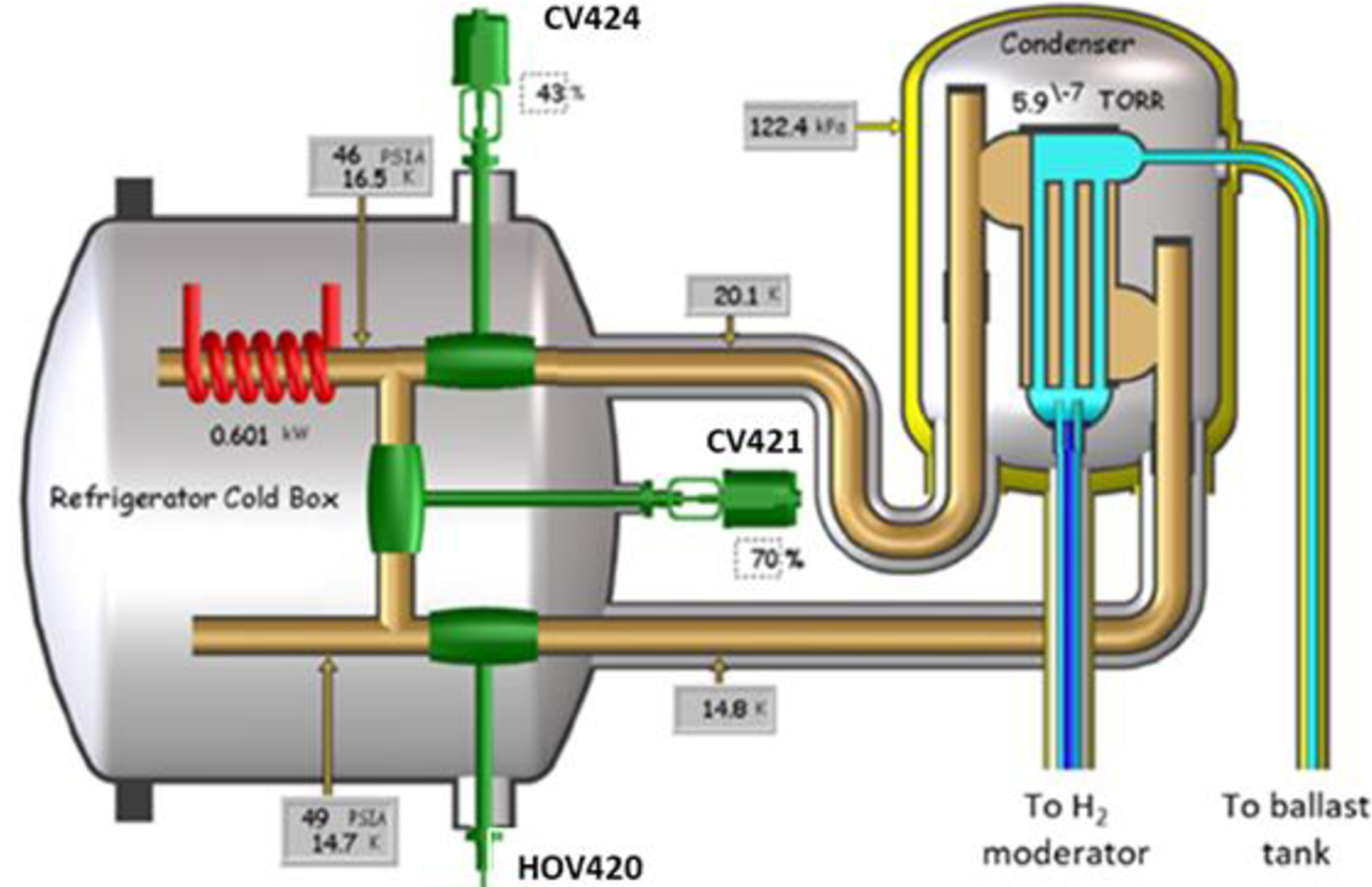
Original Unit 2 cold source configuration

The Unit 2 cold source hydrogen pressure was initially controlled (prior to the installation of Peewee) using Proportional Integral Derivative (PID) control of valve CV424 (see figure 3). CV424 would automatically provide more or less cooling to the condenser as the neutronic heat load changed. As the helium refrigerator capacity was greater than the Unit 2 heat load, a portion of the cold helium was bypassed internally through valve CV421 in the refrigerator cold box. The return temperature to the refrigerator was maintained at a constant 18 K, with a cold box internal heater making up the balance of heat load to maintain a constant 2.2 kW load on the refrigerator.

**Figure 4.**
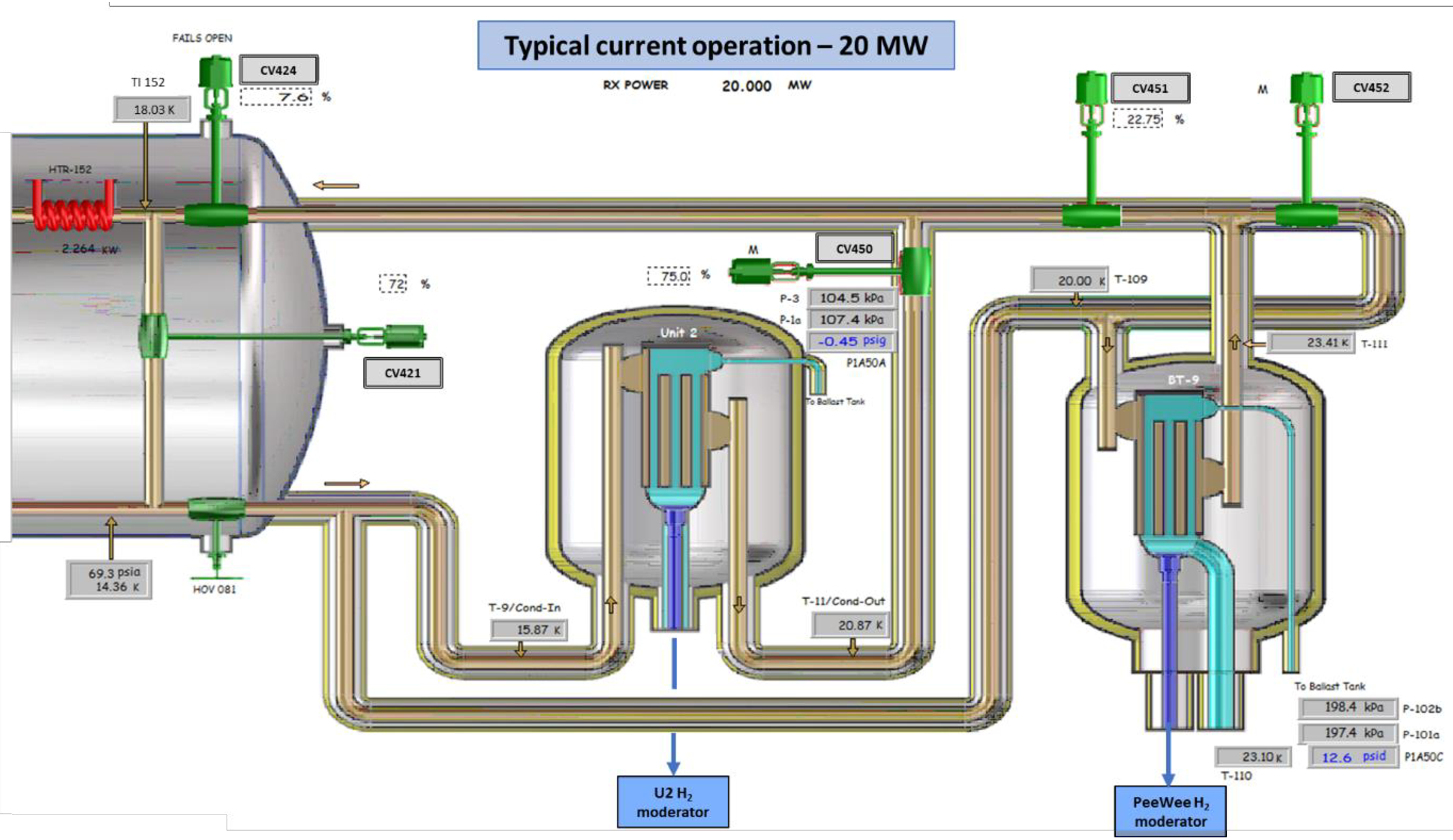
Current cold source configuration for Unit 2 and Peewee

When Peewee was installed (see figure 4), initial control of the Peewee cold source was attempted using CV451 in the same manner as was done with Unit 2 and CV450. However, this did not work well as changes in flow to Peewee would cause Unit 2 control valve CV450 to overreact.

Consequently, CV450 was set at a constant 75 % open, cold box load return valve CV424 used with PID to control Unit 2 hydrogen pressure and CV451 used with PID to control Peewee hydrogen pressure. This reduced Unit 2 hydrogen pressure oscillations but did little for Peewee hydrogen pressure oscillations. After some period of observation, it was determined that if the CV451 minimum value setpoint were increased, overall oscillations could be reduced. This change worked relatively well, except that slight changes in the system could result in renewed oscillations or hydrogen pressure decrease if the CV451 minimum value was not adjusted manually.

Since the new 7 kW refrigerator was commissioned, a more stable configuration has been programmed. Keeping records on the variations and how we changed the CV451 minimum during reactor startups and rundown recoveries, a new control routine was implemented which includes 6 or 7 decisions on whether to increase or decrease the minimum setpoint for CV451. The control routine uses the following logic: Peewee hydrogen pressure minimum, maximum, differential and average are monitored for a 5 min period. Based on these four values, the program logic slightly increases or decreases control valve CV451 minimum setting. This method has an advantage, in that the PID stays in automatic mode with no operator intervention required. If hydrogen pressure goes high (which could result in a reactor rundown), CV451 position is changed as needed to recover the pressure to the desired value of 200 kPa. Recently, the same control algorithm was implemented with load return valve CV424 with similar improved results for Unit 2 (automatically adjusting minimum setpoint with significant decrease in valve oscillations). It also successfully controls system pressures while remaining in automatic, even during transients like reactor rundowns. One difference with the control of this valve is that when reactor power is below 5 MW, CV424 minimum setpoint is 3.5 %. This is to assure that there is always some flow to the cold sources to prevent temperature spikes and possible damage to the cold sources. When the reactor is at nominal power (20 MW), CV424 minimum setpoint is initially set to 8.8 %, and allowed to slowly adjust by comparing the Unit 2 hydrogen minimum and maximum for the same 5 min period.

## 6. Design challenges – future operation with LD_2_/LH_2_

Future operation of the deuterium cold source requires operating the helium cryoplant at a higher supply temperature. Deuterium melts and vaporizes at a higher temperature than hydrogen. Operating the helium cryoplant at the current 14 K supply temperature risks freezing deuterium in the D_2_ cold source condenser. Table 2 shows the thermodynamic properties and physical geometry for the current and planned cold sources.

Although the deuterium cold source requires a higher helium supply temperature, the Peewee cold source will continue to operate with hydrogen. If it were filled with LD_2_, the neutron beam intensity would be reduced by at least a factor of two. This could be mitigated somewhat by using a larger volume of LD_2_. However, as the moderator diameter is fixed by BT-9 geometry, a longer moderator vessel would be required. A longer vessel would place the moderator farther from the reactor core, with a resulting decrease in neutron flux, as flux decreases with distance from the core.

Peewee currently operates with the hydrogen system pressure approximately 2 bar. This higher operating pressure allows the Peewee thermosiphon to function at a higher temperature of approximately 22.9 K, as the liquid/vapor saturation temperature is directly proportional to pressure.

Future operation of the cold sources with a single helium supply temperature is desirable in that it would require no modifications or additions to the current helium cooling system. *The challenge is to supply helium to the condensers at a temperature and mass flow rate that will prevent freezing of the deuterium and still provide liquefaction of the hydrogen*. The helium *inlet temperature* to the condensers must be *low enough* so that as heat is transferred from the hydrogen to the helium in the condenser heat exchanger, the helium temperature does not rise above the hydrogen liquid saturation temperature. If this were to happen, hydrogen would stop condensing and the condenser thermosiphon stop working.

Additionally, the helium *flow rate* to the condenser must be *high enough* to absorb the heat from the hydrogen without the helium temperature rising above the hydrogen liquid saturation temperature. That is: the heat absorbed by the helium is dependent on both the mass flow rate and temperature rise across the condenser heat exchanger.

**Figure 5.**
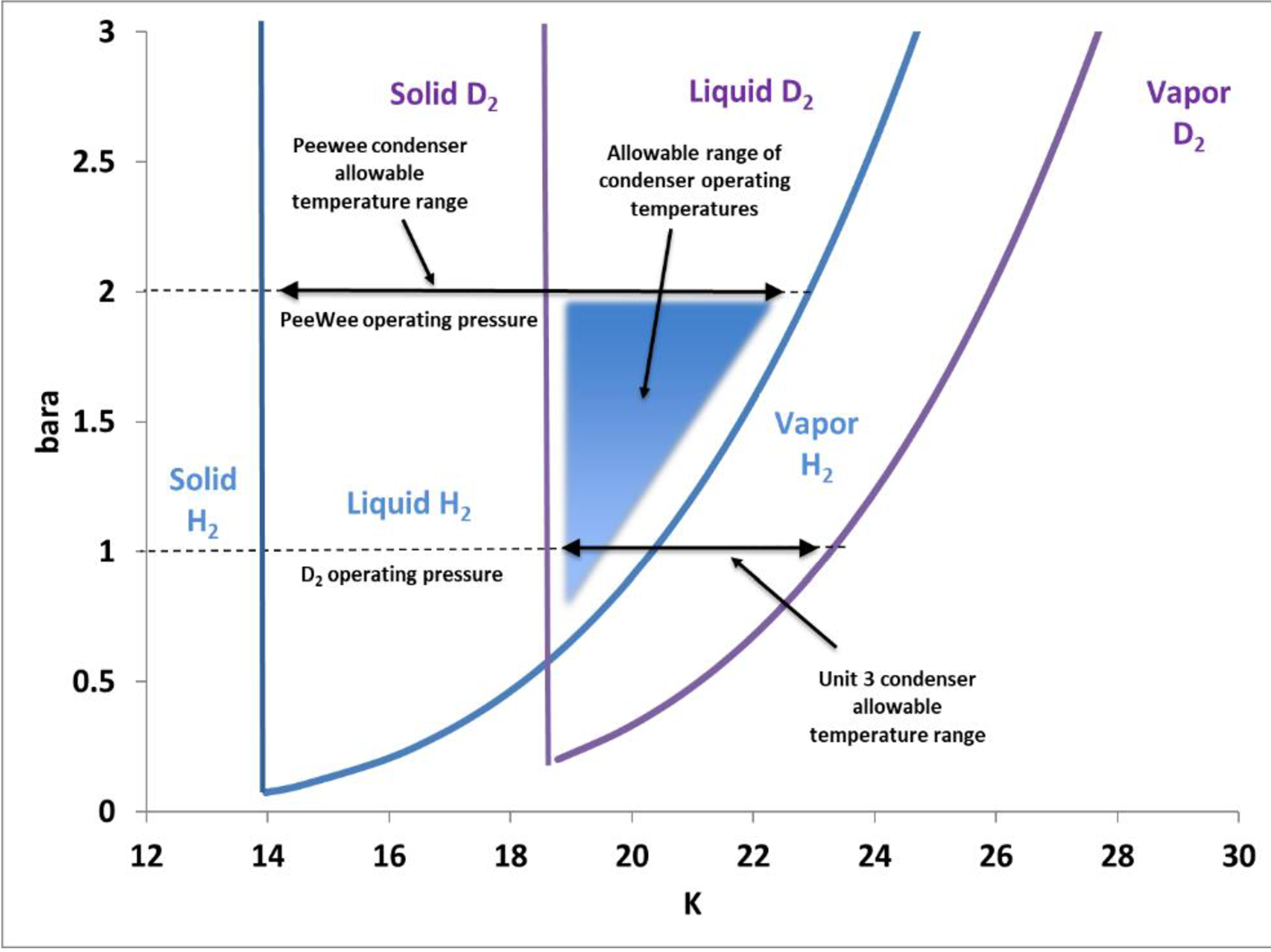
Allowable inlet temperature range to D_2_ and Peewee cold sources

Figure 5 shows the expected operating pressures and acceptable temperature limits of the deuterium and Peewee cold sources.

## 7. Observation from current operations

The new 7 kW helium refrigerator provides cooling for the current LH_2_ cold sources. Even when operating at ≈ 4 kW, it is oversized for the current heat loads, with a portion of the helium flow bypassed through valve CV421 in the cold box. Although total helium mass flow is measured, there is no direct measurement of flow through the individual Unit 2, Peewee and bypass helium circuits. Estimates of flow through each circuit can only be made based on valve characteristics, measured temperatures and pressures, and estimated heat loads. During normal operation, helium is supplied from the cold box at approximately 14 K. Silicon diode temperature sensors measure cold box helium outlet temperature and Unit 2 and Peewee condenser inlet and outlet temperatures. We observe that during normal operation, the condenser outlet temperatures are typically ≈ 0.5 K higher than the condenser hydrogen saturation temperature as calculated from the hydrogen system pressures. We also observed that there appears to be approximately a 1.5 K increase in temperature between the cold box and the Unit 2 condenser, and a 4 K increase in temperature between the cold box and the Peewee condenser. It was assumed that this temperature increase was due to static heat leak into the helium piping between the cold box and cold source condensers. This observation raised the concern that if the helium from the cold box were supplied at a temperature sufficient to liquefy D_2_ in the new deuterium condenser, the apparent helium temperature increase to Peewee would result in a temperature too warm to liquefy H_2_ in the Peewee condenser. We note that although the condenser helium outlet temperatures tracked well with hydrogen system saturation temperature, the helium inlet temperatures were not always as expected based on estimated flow rates and heat leaks into the vacuum jacketed piping.

## 8. Options to consider for LD_2_ upgrade

Based on observed operational data, NCNR is evaluating a number of options to mitigate the potential risks associated with operation of the new deuterium and existing Peewee cold sources in parallel.

### 8.1. Hardware changes considered for LD_2_ upgrade

NCNR is currently evaluating the following options to address the temperature mismatch risk between LD_2_ and LH_2_

- Additional cooling – If Peewee condenser inlet temperature is too high, there are several options to provide a secondary cooling source to that stream. The 3.5 kW helium refrigerator that was replaced with the new 7 kW refrigerator is still installed at the NBSR but is currently idle. The Peewee cold source could be completely separated from the new deuterium cold source, and cooling supplied by the recommissioned 3.5 kW refrigerator. Alternately, other smaller cryocoolers (Stirling or G-M) could be installed in line upstream of the Peewee condenser to provide a few degrees of cooling, sufficient to assure hydrogen condensation in the condenser.
- Additional heating – This option would require operating the refrigerator at a colder temperature to assure hydrogen liquefaction at the Peewee condenser and installing a small heater upstream of the deuterium condenser to assure the helium supply temperature was low enough to condense deuterium, but warm enough to prevent deuterium freezing in the condenser. Preliminary calculations estimate that a 1.5 kW heater would be sufficient.
- Change Peewee operating point – Peewee cold source hydrogen operating pressure is currently 200 kPa, 22.9 K. Increasing the Peewee hydrogen pressure to 350 kPa would result in an operating temperature of 25.4 K. This higher operating temperature could eliminate the need for cooling the condenser helium supply. However, this would require recertifying the Peewee hydrogen system to a higher maximum allowable pressure.
- Replacing VJ flex hose with hard VJ piping – There is some concern that a portion of the vacuum jacketed helium piping to the Peewee condenser has a larger static heat leak with a higher helium temperature. Replacing this hose with solid vacuum jacketed pipe would minimize this heat leak. Installation of the new LD_2_ cold source will require rework of this piping, so replacing the flexible portion with hard pipe is likely.

### 8.2. Operational tests with current configuration to mitigate risk

Before making any costly hardware changes to the cold sources, tests were planned with the existing system to better understand system parameters and help in deciding the best design options to pursue. Tests were planned to operate the helium refrigerator with a 17 K supply temperature to the cold sources. This temperature was based on the assumption that 17 K at the cold box would result at 18.5 K at the deuterium condenser and 21 K at the Peewee condenser – warm enough to liquefy deuterium and still cold enough to liquefy hydrogen at Peewee.

## 9. Progress to date

### 9.1. Operational tests

An initial test was performed with a 17 K helium supply temperature and no power to the reactor (i.e. the helium circuit cooled only the cold source static heat load). During this test both cold source condenser thermosiphons functioned normally. Based on those results, an additional short test was performed with the 17 K helium supply to the cold sources and the reactor power at 1 MW. This test demonstrated although there were some oscillations in the control valves and system pressures, both Unit 2 and Peewee cold source thermosiphons operate stably.

A final test was performed with a 17.5 K helium supply from the cold box, and reactor power as high as 20 MW. For this test, the Unit 2 cold source was operated at a hydrogen system pressure of ~200 kPa. This was done so that the Unit 2 hydrogen saturation temperatures would approximate LD_2_ liquid saturated temperature at 100 kPa (the expected operating pressure of the LD_2_ cold source). During this test, both cold source thermosiphons also operated stably.

**Figure 6.**
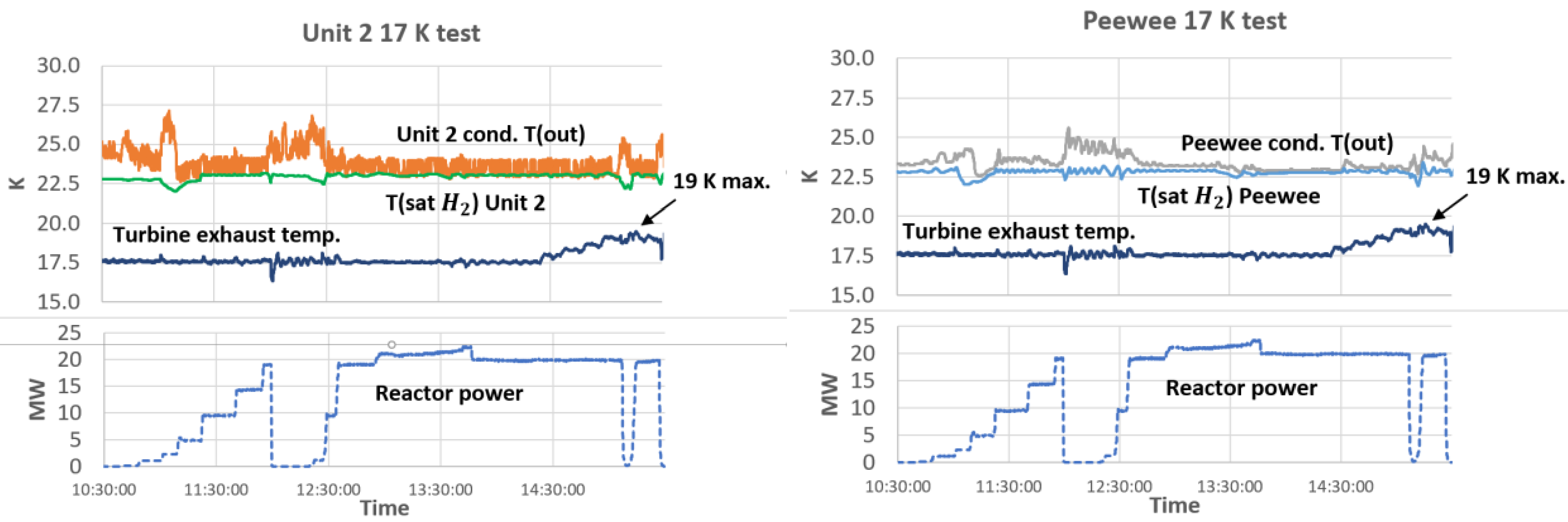
Unit 2, Peewee condenser He outlet temperatures and reactor power

Based on the uncertainty of condenser helium inlet temperatures, a concern was raised about the risk of freezing deuterium with a helium supply temperature from the cold box of 17 K (as deuterium freezes at ≈ 18.5 K at 100 kPa). To address this concern, during the final test the cold box supply temperature was raised in steps to ~ 19 K with the reactor at 20 MW. At this condition, both Unit 2 and Peewee cold source thermosiphons continued to function properly. The reactor was run down, and power reduced to 200 kW and then increased back to 20 MW. During the rundown and recovery to 20 MW, the cold sources continued to function properly with no trips or alarms. figure 6 shows stable condenser temperatures for all reactor powers up to 20 MW, and turbine exhaust temperature from 17.5 K to 19 K maximum.

### 9.2. Hardware design and fabrication

A preliminary design for the cryostat assembly, consisting of the moderator chamber, vacuum jacket, helium containment and a heavy water-cooling jacket, has been completed. A 16 m^3^ ballast tank and a pair of condensers (one spare) have been procured and delivered. A cold shock to 15 K has been completed on one condenser assemblies. Contracts for the design of the cryostat assembly and prototype vessel were awarded in 2018. It is expected that installation of the LD_2_ source will be initiated in 2023.

## 10. Summary

1. Tests performed have shown that both the Unit 2 and Peewee cold sources can operate with a turbine exhaust helium temperature as high as 19 K. At 19 K supply, the cold sources successfully recover from a reactor rundown without trips or alarms to the cryogenic system.
2. Based on test results, it is reasonable to conclude that we can eliminate the option of using the old 3.5 kW refrigerator, or other cooling options for Peewee (Stirling or G-M). We should still hold open the options of operating Peewee at a higher pressure if necessary and consider a small heater for the LD_2_ condenser to minimize risk of freezing deuterium in the condenser heat exchanger.
3. To reduce uncertainty in measuring system parameters, we should consider adding flowmeters and improved temperature sensor measurement when upgrading to the new LD_2_ cold source.
4. Overall progress on the deuterium upgrade project is on track. Condensers and a new ballast tank have been received, and work continues on the manufacture of prototype cold source cryostats.
5. Current plans for the idle 3.5 kW refrigerator are to relocate it to the National Aeronautics and Space Administration (NASA) Kennedy Space Center in Florida.

## Figures and Tables

**Table 1.** Neutronic cryogenic heat loads – current for LH_2_ and planned for LD_2_

Radiation Source	Unit 2		Peewee		LD_2_	

	H_2_	Al	H_2_	Al	D_2_	Al

Neutrons	104	3	33	1	440	6
Beta particles		308		29		657
Gamma rays	185	815	25	74	1053	1538
Subtotal	289	1126	58	104	1493	2111

Total cryogenic heat load (Watt)	1415		162		3604	

**Table 2.** Thermodynamic properties, Cold Source geometry.

**Cold Source**	**LH_2_ (Peewee)**	**LH_2_ (Unit 2)**	**LD_2_ (proposed)**

Operating pressure (kPa)	200	100	100 to 200
Boiling Point (K)	23.0	20.4	23.2 to 25.9
Melting Point (K)	14	13.8	18.8 to 19.0
Density (kg/m^3^)	67.5	70	164 to 157
**Geometry**	**Elliptical**	**Elliptical Annulus**	**Cylindrical**
Dimensions (cm)	11	32×24	40×40
LH_2_/LD_2_ thickness (cm)	4.5	2.3	3.2
Liquid volume (liter)	0.45	5	35
Mass (kg)	0.03	0.32	5.2
Al mass (kg)	0.14	2.8	7.2
